# Novel motorized spiral enteroscopy-assisted ERCP in a case of surgically altered anatomy

**DOI:** 10.1055/a-2132-4897

**Published:** 2023-08-21

**Authors:** Awanish Tewari, Vikram Uttam Patil, Mahesh Kumar Goenka

**Affiliations:** Institute of Gastrosciences and Liver Transplant, Apollo Multispeciality Hospitals, Kolkata, India

Endoscopic retrograde cholangiopancreatography (ERCP) in patients with surgically altered anatomy is intrinsically challenging 1 . Pooled rates of technical success, clinical success, and adverse events with balloon-assisted ERCP are reported to be 71.4 %, 58.7 %, and 8.4 %, respectively 2 . We report a case where we successfully performed novel motorized spiral enteroscopy (NMSE)-assisted ERCP in a patient with surgically altered anatomy.

A 70-year-old man with gastric diffuse large B-cell lymphoma underwent partial gastrectomy with Roux-en-Y gastrojejunostomy followed by chemotherapy 10 years prior to the current admission. He presented this time with a 6-week history of severe upper abdominal pain, jaundice, and pruritus. Evaluation showed acute mild biliary pancreatitis, cholelithiasis with choledocholithiasis, and a polypoidal growth at the right vesico-ureteric junction. Magnetic resonance cholangiopancreatography showed chronic cholecystitis with choledocholithiasis ( Fig. 1 ). We proceeded with NMSE (PSF-1; Olympus Medical Systems Corporation, Tokyo, Japan)-assisted ERCP via an antegrade route ( Video 1 ).

**Fig. 1:**
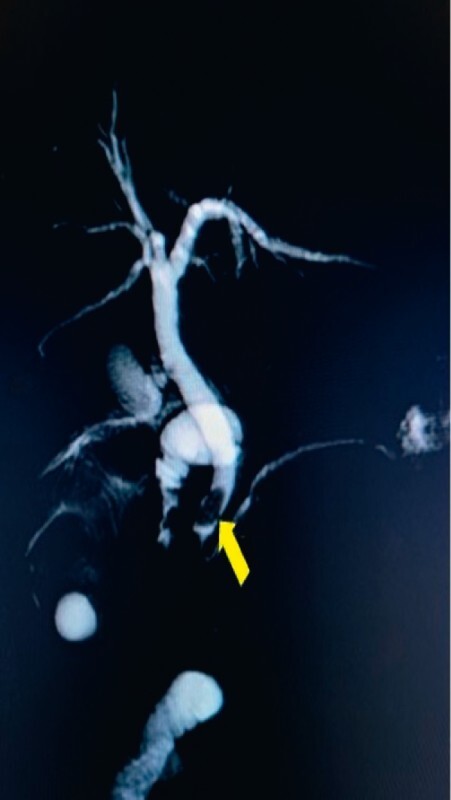
Magnetic resonance cholangiopancreatography showing choledocholithiasis, elongated and large calculus, 1.6 × 0.8 cm, in the distal common bile duct (CBD; arrow) with dilated CBD (9.2 mm) and intrahepatic biliary radicals.

**Video 1** Novel motorized spiral enteroscopy-assisted endoscopic retrograde cholangiopancreatography.


After identifying the anastomotic and jejunojenostomy sites ( Fig. 2 a ), the afferent (biliopancreatic) limb was entered. The biliary opening was noted at approximately 80 cm from the anastomosis. A triple-lumen sphincterotome was used to selectively cannulate the common bile duct (CBD) ( Fig. 2 b ). Cholangiogram revealed an oblong CBD calculus. Sphincteroplasty was performed, followed by balloon sweeps. A CBD calculus with concretions was removed ( Fig. 2 c ) and a biliary stent was deployed ( Fig. 2 d ). Total procedure duration was 40 minutes. No adverse events were noted. Jaundice resolved within a few days.

**Fig. 2:**
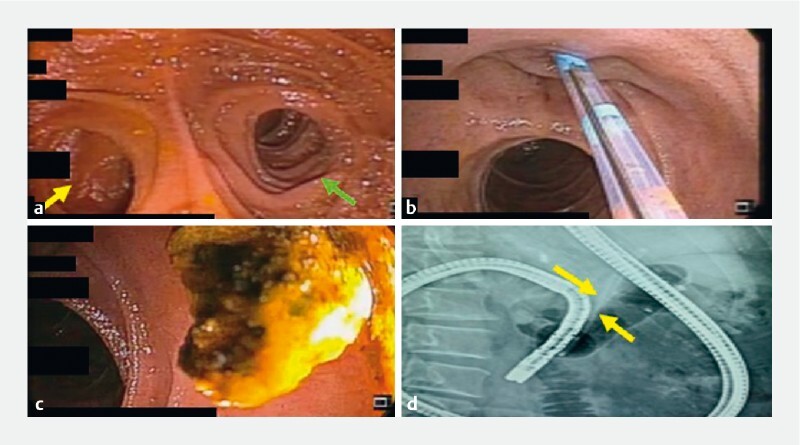
Common bile duct (CBD) clearance using motorized spiral enteroscopy-assisted endoscopic retrograde cholangiopancreatography. **a** Jejunojejunostomy site – afferent limb (green arrow) and efferent limb (yellow arrow). **b** Small periampullary diverticulum was noted, and selective CBD cannulation was performed using a triple-lumen sphincterotome. **c** Extracted CBD calculus. **d** Cholangiogram showing motorized spiral enteroscope with CBD stent (yellow arrows) after CBD clearance.

The patient underwent cystoscopy 2 days later, with transurethral resection of the bladder tumor and cystodiathermy. Biopsy revealed noninvasive papillary urothelial carcinoma. He then underwent laparoscopic cholecystectomy (histology revealed chronic cholecystitis).

After 6 weeks, NMSE-assisted ERCP was repeated and the CBD stent removed. The patient recovered well and was discharged.

In surgically altered anatomy, the normal ERCP procedure has limited success. NMSE-assisted ERCP can make the procedure more accessible.

Endoscopy_UCTN_Code_TTT_1AR_2AH and Endoscopy_UCTN_Code_TTT_1AP_2AD

## Notes

Endoscopy E-Videos

https://eref.thieme.de/e-videos
*E-Videos* is an open access online section of the journal *Endoscopy* , reporting on interesting cases and new techniques in gastroenterological endoscopy. All papers include a high-quality video and are published with a Creative Commons CC-BY license. Endoscopy E-Videos qualify for HINARI discounts and waivers and eligibility is automatically checked during the submission process. We grant 100% waivers to articles whose corresponding authors are based in Group A countries and 50% waivers to those who are based in Group B countries as classified by Research4Life (see: https://www.research4life.org/access/eligibility/ ). This section has its own submission website at https://mc.manuscriptcentral.com/e-videos
